# Gender Specific Association of RAS Gene Polymorphism with Essential Hypertension: A Case-Control Study

**DOI:** 10.1155/2014/538053

**Published:** 2014-04-17

**Authors:** Kh. Dhanachandra Singh, Ajay Jajodia, Harpreet Kaur, Ritushree Kukreti, Muthusamy Karthikeyan

**Affiliations:** ^1^Department of Bioinformatics, Alagappa University, Karaikudi, Tamil Nadu 630 004, India; ^2^Genomics and Molecular Medicine Unit, CSIR-Institute of Genomics and Integrative Biology, Mall Road, New Delhi 110007, India

## Abstract

Renin-angiotensin system (RAS) polymorphisms have been studied as candidate risk factors for hypertension with inconsistent results, possibly due to heterogeneity among various genetic and environmental factors. A case-control association study was conducted to investigate a possible involvement of polymorphisms of three RAS genes: *AGT* M235T (rs699), *ACE* I/D (rs4340) and G2350A (rs4343), and *AGTR1* A1166C (rs5186) in essential hypertensive patients. A total of 211 cases and 211 controls were recruited for this study. Genotyping was performed using PCR-RFLP method. The genotype and allele distribution of the M235T variant differed significantly in hypertensives and normotensives (OR-CI = 2.62 (1.24–5.76), *P* = 0.006; OR-CI = 0.699 (0.518–0.943), *P* = 0.018), respectively. When the samples were segregated based on sex, the 235TT genotype and T allele were predominant in the female patients (OR-CI = 5.68 (1.60-25.10), *P* = 0.002; OR-CI = 0.522 (0.330–0.826), *P* = 0.005) as compare to the male patients (OR-CI = 1.54 (1.24–5.76), *P* = 0.34; OR-CI = 0.874 (0.330–0.826), *P* = 0.506), respectively. For *ACE* DD variant, we found overrepresentation of “I”-allele (homozygous II and heterozygous ID) in unaffected males which suggest its protective role in studied population (OR-CI = 0.401 (0.224–0.718); *P* = 0.0009). The M235T variant of the *AGT* is significantly associated with female hypertensives and *ACE* DD variant could be a risk allele for essential hypertension in south India.

## 1. Introduction

Human essential hypertension (EHT) is a multifactorial trait with a complex genetic basis. This complex disease is due to the consequence of an interaction between various environmental and genetic factors and it plays a major role in blood pressure (BP) variation [1]. It does not follow the Mendelian mode of transmission [2]. The genetic contribution is estimated to be between 30% and 40% of BP variation [3]. In recent years, a series of genes have been proposed to influence the mechanism of blood pressure. Some evidences of association between these genes had been reported [4–6] but on the contrary these associations were not always significant [7].

More than 150 candidate genes have been implicated in the regulation of blood pressure that is linked to several pathways. Among these genes, RAS has significant direct involvement in the BP regulation as it is reported in several studies and most of the antihypertensive drugs are targeting this system. The genetic variation of RAS encoding genes, angiotensinogen (*AGT*), angiotensin-1-converting enzyme (*ACE*), and angiotensin II type 1 receptor (*AGTR1*), were associated with EHT and have been important genes for the association studies in various populations [8–10].* AGT*, the natural substrate of RAS, is synthesized in the liver and released into blood circulation. The potential role of* AGT* gene in hypertension was originally explored by Jeunemaitre group through linkage and association study in the causation of human EHT in Utah and French populations [4].* AGT* variants have been shown to associate with serum* AGT* in black and white children, providing a potential mechanism for genetic associations [11, 12]. Among the identified major molecular variants of* AGT*, M235T and T174 M variants had a significant association with hypertension [13]. But associations with these variants are found to be contradictory in different populations [14]. Two different meta-analyses in Chinese populations have also confirmed that T allele of* AGT* M235T polymorphism is associated with essential hypertension [15, 16].

*ACE* is a key zinc metalloenzyme of the RAS and is widely distributed in the kidney [17]. The* ACE* catalyzes the conversion of angiotensin I to the biologically active peptide, angiotensin II, which is involved in the control of fluid electrolyte balance and systemic blood pressure [18]. This polymorphism is characterized by the presence (insertion) or absence (deletion) of a 287 bp AluYa5 element inside intron 16. Although I/D polymorphism is located in intronic region of the* ACE* gene, several investigators have found that the D allele is related to increased activity of* ACE* in serum. The highest serum* ACE* activity was seen in the DD genotype while the lowest was seen in the II genotype [19]. Among the* ACE* gene polymorphisms of exon 17, G2350A variant has the most significant effect on plasma* ACE* concentrations as it accounts for 19% of the total variance in* ACE* plasma levels.* ACE* I/D is in linkage disequilibrium (LD) with* ACE* G2350A and both the variants are associated with essential hypertension with contradictory result [20, 21]. Meta-analyses in different studies also found that DD genotype of* ACE* I/D polymorphism [22, 23] and AA genotype of ACE G2350A polymorphism are associated with essential hypertension [24].

The* AGTR1*, a receptor for angiotensin II, is a member of the G-protein-coupled receptor super family expressed in most tissues, where receptor activation leads to vasoconstriction, water retention, and vascular smooth muscle cell proliferation and hypertrophy [25, 26]. The polymorphism A1166C in the 3′ untranslated region of the* AGTR1* gene was detected in study by Bonnardeaux et al., (1994) who also identified its association with hypertension [27]. Several recent findings and meta-analysis reported that it is associated with essential hypertension [28, 29]; however, conflicting results are also reported by few studies [30, 31].

The goal of the present analysis is to assess the effect of a combination of variants at different loci of pathophysiological pathway of RAS genes in south Indian population. In this case-control study, we examined possible associations between polymorphisms of the* AGT*,* ACE*, and* AGTR1* genes and hypertension in the south Indian population age between 30 and 70 years. We therefore determined the association between* AGT* M235T,* ACE* I/D,* ACE* G2350A, and* AGTR1* A1166C polymorphisms in the study population.

## 2. Materials and Methods

### 2.1. Study Population

Blood sample (5 mL) were collected from patients (*n* = 211) and control subjects (*n* = 211) between the age of 30 and 70 years from the clinics after informed consent form was obtained from all the participating volunteers. Patients were diagnosed in accordance with JNC 7 guidelines [32] and hypertension was defined as systolic BP (SBP) of ≥140 mm Hg and/or diastolic BP (DBP) of ≥90 mm Hg or prior diagnosis of essential hypertension by a physician or current use of antihypertensive medication or individual having a history of hypertension. All the cases included in the study were of essential hypertension as diagnosed by the physician. Cases with secondary forms of hypertension, myocardial infarction, and cerebrovascular incidents or other systemic diseases and any major illness in last six month before the sample collection were excluded. Normotensive individuals (*n* = 211) with SBP ≤ 120 mm Hg and DBP ≤ 80 mm Hg with matched age, sex, and location were selected as controls. Control individuals were also devoid of associated conditions like vascular diseases, diabetes, and other systemic diseases or under any medication and BMI of ≤25 kg/m^2^. This study was approved by Institutional Ethical Committee. Clinical data and family history were recorded in the questionnaire for all the participants. All the participants belonged to Dravidian ancestry living in Tamil Nadu, south India. All the patients' samples were collected from the Outpatients Department of Government Hospitals of Tamil Nadu, south India. Control samples were also collected from the volunteers living in same place and origin. Genomic DNA was extracted using modified Miller's protocol [33] and it was quantified spectrophotometrically by OD_260_/OD_280_ ratio. Genotyping was performed with PCR-RFLP and allele specific primer methods. Primer, restriction enzyme and PCR conditions are shown in Supplementary Table 1 in Supplementary Material available online at http://dx.doi.org/10.1155/2014/538053.

### 2.2. Statistical Analysis

Differences in the* ACE*,* AGTR1*, and* AGT* genotype frequencies between the cases and controls were compared using chi-square statistics. The odds ratio (OR) and 95% confidence interval (CI) were used as a measure of the strength of the association between genotype frequencies. Statistical significance accepted level was *P* < 0.05. The frequencies of the marker alleles were estimated by allele counting method and tested for Hardy-Weinberg equilibrium (HWE). All the statistical calculations were carried out using PLINK 1.07 [34] and STATA 11.0. Power of the study was calculated using PS-Power and sample size calculation [35].

MDR (multidimensionality reduction) analysis was performed using MDR software [36] to determine the genotypic combination that may confer high or low risk for EHT and also to determine the single most predictive genetic (gene-gene/gene-environment) model for EHT. Briefly, the MDR was comprised of two steps. First, the best combination of multifactors was chosen. Second, the combinations of genotypes are classified into high- and low-risk groups [37]. Interaction analysis was performed in the open source MDR software package (v.2.0) available at http://www.epistasis.org/ [38].

## 3. Results

Association studies of genetic polymorphisms and trait for detecting the complex diseases remain controversial. However, the association study of allele and genotype frequencies of candidate genes with the unaffected and affected subjects to understand the genetic etiology of complex human traits remains an efficient method [39–41]. Taking this method into consideration, we determined the possible association of genetic polymorphisms of RAS genes with EHT with the south Indian subjects. To the best of our knowledge, few studies have been published in RAS gene polymorphisms with the genotype and allele based association study in Indian subjects. But, large number of studies has reported association between EHT and* ACE*  [42–45] or* AGT*  [46–48] gene polymorphism but lack of haplotype based association of the RAS gene with the EHT in south Indian population. Probably this could be the first comprehensive reports on RAS gene polymorphisms with association study of genotype and haplotype in relation to EHT in south Indian subjects.

A total of 422 subjects were recruited for this study, out of which 211 subjects were cases and 211 subjects were controls. Among the patients, 109 (51.66%) are males and 102 (48.34%) are females, whereas in control subjects 130 (61.61%) are males and 81 (38.99%) are females (Table 1). The mean age of control subjects is  43.71 ± 14.17 years for males and 43.90 ± 13.57 years for females. The mean ages of male patients are 54.56 ± 13.04 years and 54.37 ± 12.25 years for females. The difference in the distribution of epidemiological features were found to be significant between cases and controls with respect to gender (*χ*² =  4.26; *P* = 0.039).

Genotype distributions of all four studied polymorphisms were compatible with HWE expectation in cases as well as in controls. Differences between cases and controls in allele/genotype frequency distributions that were observed for* AGT* M235T and* ACE* I/D polymorphism (Tables 2 and 3) with T and D allele are more prevalent in cases than in controls except for* ACE* I/D polymorphism in females. Genotype/allele distribution of* ACE* G2350A and* AGTR1* A1166C polymorphisms was similar in both cases and controls (Tables 2 and 3).

To test the association of RAS gene polymorphisms with hypertension, genotype odds ratio was calculated for all the variants reported in the table.

### 3.1. *AGT* M235T Polymorphism

The genotype distribution of M235T polymorphism was significantly different between case and control subjects. Taking the MM genotype of the* AGT* M235T polymorphism as a reference, association of the MT genotype was 2.40 (95% CI: 1.11–5.37) and TT genotype was 2.62 (95% CI: 1.24–5.76), indicating a dominant effect of the T on risk and T allele is highly associated with hypertension in our recessive model (Table 4). Allelic association does not withstand after multiple corrections (alpha value 0.05/4 = 0.013; Table 2). Furthermore, the samples were segregated for subgroup analysis on the basis of gender and T allele was associated with the female patients (OR: 0.522; 95% CI: 0.330–0.826) after applying the Bonferroni corrections (Tables 2 and 3).

### 3.2. *ACE* I/D Polymorphism

Considering I/D polymorphism, the distribution of genotypes did not differ significantly between EHT patients and controls through a slight increase in the frequency of DD (39.34%) homozygous that was observed among patients as compared to controls (31.75%) (Table 3). However, our results revealed overrepresentation of “I”-allele carriers (homozygous II and heterozygous ID) in male control subjects (Table 4) which suggests its protective role in studied population. This association was estimated as odds ratio of 0.401 with 95% CI 0.224–0.718 and withstand after multiple correction (Table 4).

### 3.3. *ACE* G2350A Polymorphism

The genotype distribution of* ACE* G2350A polymorphism did not differ significantly between EHT patients and control subjects, and the allele frequencies were in HWE in both groups (Tables 3 and 4).

### 3.4. *AGTR1* A1166C Polymorphism

Genotyping frequencies of A1166C polymorphisms were identified for all the EHT patients and control subjects. Our result shows that there is no significant difference in genotype and allelic frequencies in EHT patients and controls. Furthermore, the allele frequencies are consistent with the HWE in both cases and controls (Tables 2 and 3).

### 3.5. Analysis of Epistatic Interaction

**Figure 1:**
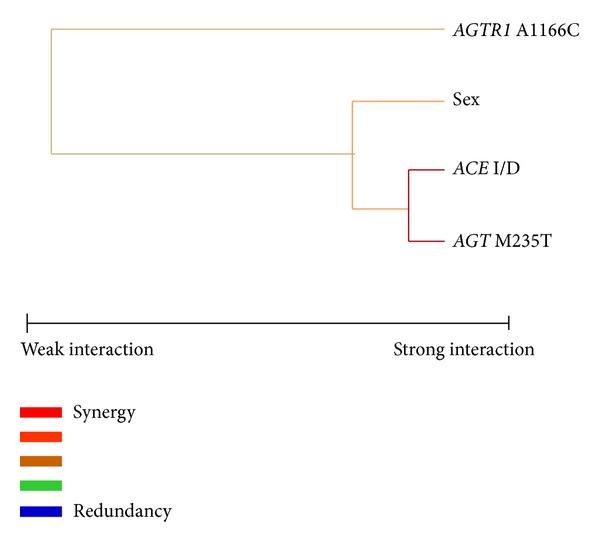
Interaction dendrogram for the four polymorphisms of RAS gene modeled by the MDR method. A red or orange line indicates synergistic or nonadditive relationship yellow line, independency, or additivity.

**Figure 2:**
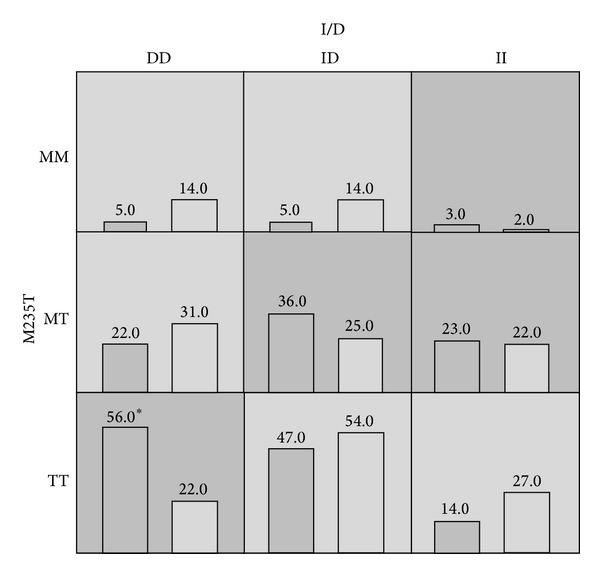
Distribution of high-risk (dark shaded) and low-risk (light shaded) genotypes among the markers studied. The summary of the distribution illustrates the hypertensives (left bars) and normotensives (right bars) for each genotype combination. Asterisk (∗) indicates the significant difference between cases and control subjects.

To elaborate the findings of the analysis, MDR analysis was applied to detect and characterize high-order gene-gene and gene-environmental interactions in cases and controls.  Figure 1 illustrates the MDR interaction information analysis of all four polymorphisms studied gene, represented in the form of a dendrogram. The MDR analysis shows synergistic effect of* ACE* and* AGT* polymorphisms in the EHT patients. To improve the quality of analysis and identify the disease risk, we have carried out combination analysis and haplotype frequencies on the basis of the polymorphism pairs selected by MDR analysis. Similar analysis between gene-environmental interactions showed moderate synergistic effect of the marker* AGT* M235T and* ACE* I/D with the sex in the development of EHT (Figure 1).  Table 5 ****summarizes the results of the MDR analysis evaluated for all possible combinations of the polymorphisms studied for the risk of developing hypertension. It shows the best model with a combination of polymorphisms in order along with its prediction error and coefficient of variation (CV) consistency. Result reveals the interaction of* AGT* M235T-*ACE* I/D polymorphisms as the overall best models with least prediction error of 0.39 and CV consistency of 10/10. High-risk (dark grey) and low-risk (light grey) genotypic combinations were determined based on the threshold value, which was 1.0 (211/211) for the present data. It was observed that TT genotype of* AGT* M235T polymorphism when present in combination with DD genotype of* ACE* I/D polymorphism conferred a 2.5 times higher risk (56/22) for developing EHT (Figure 2).

## 4. Genetic Power Test

We estimated the genetic power using the M235T polymorphism as an example; an 80% power should have to detect linkage between hypertension and T allele at type I error of 0.05 when the sample includes 211 cases and 211 controls. We also performed post hoc exploratory analyses to examine the relationships of the polymorphisms with cases and control subjects. Genetic power estimation showed that 211 cases and 211 controls had >80% power to detect linkage between M235T variant and hypertension in south Indian population.

## 5. Discussion

The renin-angiotensin system is a major endocrine/paracrine system that regulates blood pressure (BP) in our body [49], genes encoding components of this system have been strong candidates for the investigation of the genetic basis of essential hypertension and major targets for antihypertensive drugs [50]. However, previous studies in south India mainly focus on limited gene of RAS [47, 48, 51]; thus we carried out a case-control study to systemically investigate the association between polymorphisms in RAS genes and essential hypertension. The present study identifies gender specific genetic variants in RAS genes that may play crucial roles in BP regulation and susceptibility for hypertension. A significantly higher frequency of the* AGT* 235TT was observed in female patients when compared to the female controls.* ACE* DD was more prevalent in male patients when compared with male controls. So,* AGT* M235T was associated with EHT in females and* ACE* I/D was associated with males in our study population. Association of T variant with the essential hypertension in our study has shown agreement with some studies.

Jeunemaitre et al., 1992 [4], were the first to report the linkage of the molecular variants M235T with hypertension in the Caucasians. Subsequent studies among the UK/Chinese/Malaysian/south Indian supported the former finding [15, 40, 47, 48, 52] while a study of Germany/North India is inconsistent with the previous report  [47, 53]. The association studies in the Africans/African-Americans mostly found a negative association [54, 55], but the high frequency of T allele was observed in this population and it is associated with increase in plasma and mRNA* AGT* concentration [11, 55, 56].

The frequency of the 235T variant (major allele in our study) was 0.746 for this study among cases and this frequency was similar to study already reported in Chinese (0.73) and Hong Kong Chinese (0.82) population [16, 57]. The frequency of 235T variant in the 1000 genome project was found to be 0.87 in Africans, 0.64 in Americans, and 0.84 in Asians but the frequency was deviated in Europeans with the frequency of 0.41 [58]. Several studies in south India has reported that the 235T frequencies are in the range of 0.56–0.81 in the patients [13, 59–61]. But in north India the frequency was reported to be 0.31 in the cases [44]. Prasad and colleague have performed a comparative study of north and south India in control subjects and 235T variant frequency was found to be 0.33 and 0.52, respectively [62]. These overall results show that 235T variant is predominantly present in south India compared to north India. Our study showed a higher OR 2.62 (95% CI, 1.24–5.76; *P* = 0.006) compared to a recent meta-analysis [15] in Han Chinese population which reports high association between* AGT* M235T polymorphism and hypertension (OR = 1.54; 95% CI, 1.16–2.03; *P* = 0.002). On the contrary, another meta-analysis of 126 studies by Sethi et al. (2003) has reported that odds ratio for hypertension was 1.19 (1.10 to 1.30) in TT individuals in white subjects and 1.60 (1.19 to 2.15) in Asian subjects and it was not associated with SBP or DBP [63]. Instead, M235T genotype was associated with a stepwise increase in angiotensinogen levels in white subjects and a corresponding increase in risk of hypertension in both White and Asian subjects [63]. In another meta-analysis, the frequencies of the AGT T allele were 80% in cases and 72% in controls. The pooled OR (with 95% CI) of TT versusMT + MM was 1.76 (1.44–2.16) (*P* < 0.00001) with T versus M of 1.54 (1.31–1.81). The pooled OR of MM versus MT + TT was 0.67 (0.45–1.00) (*P* = 0.05) [16].

In this study, the significantly higher prevalence of 235T allele in female patients is in agreement with the previous report that the T allele was significantly more prevalent among female ETH than in control subjects [4]. In contrast, Freire et al. (1998) [64] found that the* AGT* M235T homozygous mutation genotype was significantly higher in males compared to females. Earlier studies have found that plasma* AGT* levels in postmenopausal women are slightly higher [65] than in men [66]. Plasma angiotensinogen levels are more increased in M235T variant than the 235 M variant with administration of ethinyl estradiol (EE), a synthetic estrogen. The* AGT* G (-6) variant is less transcriptionally active with lower levels of* AGT* mRNA [16, 67] and a significant linkage disequilibrium between M235T and G-6A was also reported. These findings reveals that G-6A polymorphism, which is linked to nonfunctioning M235T, increased the plasma* AGT* level via regulation of* AGT* gene transcription and was involved in the pathogenesis of the predisposition to hypertension. Thus, sex hormones such as estrogen might bind to the core promoter region and enhance the transcription of* AGT* gene. However, it is also can be hypothesized that estrogen may bind to the -6A variant more effectively and* AGT* gene expression could be more in this variant. Hence, the angiotensinogen promoter is directly controlled by estrogen [68, 69].

The I/D allelic variant (intronic deletion of a 287 bp Alu sequence repetitive element, D allele) is one of the most intensively investigated genetic polymorphism in the field of hypertension and cardiovascular disease research [70, 71]. In the present study, dominant model of* ACE* gene in male (II + ID versus DD) is associated with EHT in south Indian and it was also observed that I allele has a protective role. Overrepresentation of D allele in male cases represents strong association in pathogenesis of hypertension in male. This is in accordance with the previous report by many studies [72–74]. Our study showed a lower OR 0.401 (95% CI 0.224–0.718; *P* < 0.01) compared to a recent meta-analysis [22] in whole Chinese population which reports high association with* ACE* DD polymorphism with hypertension (OR = 1.27; 95% CI 1.17–1.46; *P* < 0.01). The D allele of* ACE* was initially suggested to be associated with increased levels of serum* ACE* activity as compared with I variant [75, 76]. In contrast to this, Zee et al. (1992) has reported that “I” allele was associated with high blood pressure in an Australian population with strong evidence of familial hypertension [77]. In recent meta-analysis, the distribution of the D allele frequency was 0.45 for the EH group and 0.40 for the control group. The summary OR for the distribution frequency of D allele was 1.27 (5% CI 1.17–1.37). The heterogeneity among the 67 studies was also significant (*P* < 0.00001,* I*(2) = 71.4%). There was a significant association between distribution frequency of the D allele and EH risk in Han, Kazakh, Tibetan, Zhuang, and unclassified nationalities (*P* < 0.05). Contradictory result was observed in the national minorities, such as Mongolian, Uighur, Yi, Dongxiang, Yugu, Korean and Gamel, and the* D* allele; and EH risk was not significant (*P* > 0.05) [22].

The mechanism of the sex specific association with hypertension remains unclear. One notion is that estrogen protects against hypertension. Even though estrogen upregulates the* AGT* gene expression but it again acts as negative feedback mechanism in controlling renin secretion and then the* ACE* secretion in RAS [78]. Sex differences exist in the regulation of arterial pressure and renal function by RAS. This may in part stem from a differential balance in the pressor and depressor arms of the RAS (Hilliard et al., 2013). In males, the* ACE*/AngII/*AGTR1* pathways are enhanced, whereas, in females, the balance is shifted towards the* ACE2*/Ang(1-7)/MasR (Mas receptor) and angiotensin type 2 receptor (AT_2_R) pathways [79]. Studies reported that premenopausal women, as compared to age matched men, are protected from renal and cardiovascular disease, and this differential balance of the RAS between the sexes likely contributes [80, 81].

There were some limitations in this study. First, the age and sex match case control was not available in the sample studied. Our approach is based on a sample with a relatively homogeneous genetic background, and therefore the results are unlikely to be affected by unmeasured confounding factors of population stratification. This study did not measure central obesity and psychiatric comorbidities, the major risk factor for hypertension. There are no measurements of plasma angiotensinogen levels or other markers of RAS activation available to correlate directly with the genetic polymorphisms investigated in this study.

## 6. Conclusion

In summary, the genotype distributions of the* AGT* M235T polymorphism influenced the risk of essential hypertension in south Indian women and* ACE* DD is a risk in south Indian male population. MDR analysis reveals that if both TT and DD genotypes are present, prevalence of EHT is higher in the present study. The haplotype based MDR analysis suggests that we had adequate power to detect the functional relationship of the best factor model, increasing the risk of essential hypertension associated with combined genetic variations. This is the first report to evaluate the simultaneous association of* ACE*,* AGTR1*, and* AGT* gene polymorphisms in essential hypertension by haplotype based analyses and gender specific association in south India. Future studies are required to consider the joint effects of several candidate genes to dissect the genetic framework and gender specific association of essential hypertension.

## Supplementary Material

Supplementary Table 1: Details of the oligonucleotides for amplification and screening for four RAS polymorphisms using allele specific primer and PCR-RFLP methods.

## Figures and Tables

**Table 1:** Sample size, sex ratio, and basic characteristics (means, standard deviation) of study sample.

	Cases (*n*)	Controls (*n*)
*N*	211	211
Male	109	130
Female	102	81
Age (years), male	54.56 ± 13.04	43.71 ± 14.17
Age (years), female	54.37 ± 12.25	43.90 ± 13.57

*N*: sample size.

**Table 2:** Distributions of allele frequencies in cases and controls stratified gender wise.

Gene	Allele	Minor allele	Freq. minor allele case	Freq. minor allele control	*χ* ^2^	OR	*P* value
*AGT* M235T	M/T	M	0.254	0.327	5.527	0.699 (0.518–0.943)	0.018*
Female			0.226	0.358	7.797	0.522 (0.330–0.826)	0.005*
Male			0.280	0.308	0.443	0.874 (0.588–1.299)	0.506
*ACE* I/D	I/D	I	0.398	0.462	3.524	0.770 (0.586–1.012)	0.060
Female			0.441	0.420	0.169	1.091 (0.719–1.656)	0.681
Male			0.357	0.489	8.265	0.584 (0.404–0.844)	0.004*
*ACE* G2350A	G/A	A	0.173	0.164	0.136	1.070 (0.746–1.535)	0.713
Female			0.172	0.210	0.866	0.780 (0.461–1.318)	0.352
Male			0.174	0.135	1.444	1.357 (0.824–2.236)	0.229
*AGTR1* A1166C	A/C	C	0.384	0.401	0.244	0.933 (0.708–1.230)	0.622
Female			0.363	0.414	0.985	0.807 (0.529–1.233)	0.321
Male			0.404	0.392	0.064	1.049 (0.726–1.515)	0.800

*χ* ^2^: Chi-square with 1 degree of freedom; OR: odds ratio; *P < 0.05: statically significant (alpha value 0.05/4).

**Table 3:** Distribution of genotype frequencies of RAS gene polymorphisms in patients and control subjects.

SNPs	Genotype	Case % (*N* = 211)	Control % (*N* = 211)	OR	95% CI	*P* value
*AGT * M235T	MM	6.16	14.22	Ref		
	MT	38.39	36.97	2.40	1.11–5.37	0.016
	TT	55.45	48.82	2.62	1.24–5.76	0.006*
Male	MM	8.26	12.31	Ref		
	MT	39.45	36.92	1.59	0.59–4.52	0.320
	TT	52.29	50.77	1.54	0.58–4.25	0.340
Female	MM	3.92	17.28	Ref		
	MT	37.25	37.04	4.43	1.21–20.09	0.011
	TT	58.82	45.68	5.68	1.60–25.10	0.002*

*ACE * I/D	II	18.96	24.17	Ref		
	ID	41.71	44.08	1.21	0.71–2.07	0.467
	DD	39.34	31.75	1.58	0.90–2.76	0.087
Male	II	17.43	23.08	Ref		
	ID	36.70	51.54	0.94	0.45–2.02	0.870
	DD	45.87	25.38	2.39	1.09–5.27	0.020
Female	II	20.59	25.93	Ref		
	ID	47.06	32.10	1.85	0.80–4.28	0.117
	DD	32.35	41.98	0.97	0.42–2.25	0.940

*ACE * G2350A	GG	71.09	72.51	Ref		
	GA	23.22	22.27	1.06	0.65–1.73	0.793
	AA	5.69	5.21	1.11	0.43–2.88	0.805
Male	GG	71.56	77.69	Ref		
	GA	22.02	17.69	1.51	0.42–5.66	0.472
	AA	6.42	4.62	1.35	0.67–2.71	0.359
Female	GG	70.59	64.20	Ref		
	GA	24.51	29.63	0.75	0.37–1.54	0.400
	AA	4.90	6.17	0.72	0.16–3.32	0.620

*AGTR1 * A1166C	AA	41.71	40.28	Ref		
	AC	39.81	39.34	0.98	0.62–1.53	0.917
	CC	18.48	20.38	0.88	0.50–1.53	0.622
Male	AA	37.61	40.77	Ref		
	AC	44.04	40.00	1.03	0.47–2.24	0.540
	CC	18.35	19.23	1.19	0.65–2.19	0.930
Female	AA	46.08	39.51	Ref		
	AC	35.29	38.27	0.79	0.39–1.61	0.484
	CC	18.63	22.22	0.72	0.30–1.70	0.409

*N*: sample size; OR: odds ratio; CI: confidence interval; Ref: reference; *P < 0.05: statically significant.

**Table 4:** Distribution of RAS gene polymorphisms (dominant and recessive model) in patient and control subjects.

SNPs	Model	TEST	Case (*N*)	Control (*N*)	OR	95% CI	*P* value
*AGT* M235T	MM + MT versus TT	DOM	94/117	108/103	0.766	0.513–1.144	0.173
	MM versus MT + TT	REC	13/198	30/181	0.396	0.184–0.813	0.006*
Male	MM + MT versus TT	DOM	52/57	64/66	0.941	0.547–1.616	0.814
	MM versus MT + TT	REC	9/100	16/114	0.641	0.239–1.624	0.308
Female	MM + MT versus TT	DOM	42/60	44/37	0.589	0.313–1.106	0.077
	MM versus MT + TT	REC	04/98	14/67	0.062	0.015–0.185	0.000

*ACE* I/D	II + ID versus DD	DOM	128/83	144/67	0.718	0.471–1.092	0.104
	II versus ID + DD	REC	40/171	51/160	0.734	0.447–1.201	0.193
Male	II + ID versus DD	DOM	59/50	97/33	0.401	0.224–0 .718	0.0009*
	II versus ID + DD	REC	19/90	30/100	0.704	0.349–1.397	0.282
Female	II + ID versus DD	DOM	69/33	47/34	1.513	0.789–2.898	0.180
	II versus ID + DD	REC	21/81	21/60	0.741	0.350–1.569	0.394

*ACE* G2350A	AA + AG versus GG	DOM	61/150	58/153	1.073	0.686–1.677	0.746
	AA versus AG + GG	REC	12/199	11/200	1.096	0.432–2.813	0.830
Male	AA + AG versus GG	DOM	31/78	29/101	1.384	0.738–2.596	0.276
	AA versus AG + GG	REC	7/102	6/124	1.418	0.394–0.273	0.540
Female	AA + AG versus GG	DOM	30/72	29/52	0.747	0.383–1.462	0.358
	AA versus AG + GG	REC	05/97	05/76	0.784	0.174–0.542	0.707

A1166C	CC + CT versus TT	DOM	123/88	126/85	0.943	0.628–1.416	0.767
	CC versus CT + TT	REC	39/172	43/168	0.886	0.530–1.477	0.623
Male	CC + CA versus AA	DOM	68/41	77/53	1.142	0.656–1.992	0.619
	CC versus CA + AA	REC	20/89	25/105	0.944	0.464–1.903	0.862
Female	CC + CA versus AA	DOM	55/47	49/32	0.764	0.405–1.439	0.373
	CC versus CA + AA	REC	19/83	18/63	0.801	0.365–1.767	0.548

DOM: dominant model; REC: recessive model; *N*: sample size; OR: odds ratio; CI: confidence interval; *P < 0.05: statically significant.

**Table 5:** Summary of MDR analysis. MDR analysis reveals that combination of *ACE* I/D and *AGT* M235T could be a high risk for the prevalence of the EHT.

Model	Training bal. acc. (%)	Testing bal. acc. (%)	Cross-validation consistency	Prediction error
Sex	0.550	0.528	08/10	0.450
I/D, M235T	**0.613**	**0.595**	**10/10**	**0.387**
I/D, M235T, and A1166C	0.655	0.569	6/10	0.345
I/D, M235T, A1166C, and sex	0.710	0.602	7/10	0.290
