# Supplementary material for: Gender Specific Association of RAS Gene Polymorphism with Essential Hypertension: A Case-Control Study
Source: Biomed Res Int. 2014 Apr 17;2014:538053. doi: 10.1155/2014/538053 (PMC4016835; doi:10.1155/2014/538053)
Supplement: Supplementary file 1 — Supplementary Table 1: Details of the oligonucleotides for amplification and screening for four RAS polymorphisms using allele specific primer and PCR-RFLP methods. [file 538053.f1.pdf]

SUPPLEMENTARY INFORMATION

Supplementary Table 1. Details of the oligonucleotides for amplification and screening for four RAS polymorphisms using allele specific primer and PCR-RFLP methods.

SNPs	F:Forward primer<br>R:Reverse primer	PCR cycling conditions (sec/min)	Restriction endonuclease	PCR product (bp)	Restriction fragment size (bp)	References
AGT M235T	F:TGACAGGATGGAAGACTGGCTGCTCCCTGC<br>R:AGCAGAGAGGTTTGCCTTACCTCTG	Tm =60°C for 45s	*Msp I*	104 bp	73, 31	[1]
ACE II	F: GCCCTGCAGGTGTCTGCAGCATGT<br>R: GGATGGCTCTCCCCGCCTTGTCTC	Tm =56ºC for 45s	PCR 287 bp Alu insertion in intron 16	DD: 319 and II: 97		[2]
ACE DD	F: TGGGACCAGAGCGCCCGCCACTAC<br>R: TCGCCAGCCCTCCCATGCCCATAA	Tm= 76 °C for 30s		II: 335 DD: no product		
ACE G2350A	F:CTGACGAATGTGATGGCCGC<br>R: TTGATGAGTTCCACGTATTTCG	Tm=58°C for 30s	*BstUI*	122	100, 22	[3]
AT_1R A1166C	F:AATGCTTGTAGCCAAAGTCACCT<br>R: GGCTTTGCTTTGTCTTGTTG	Tm=60°C for 1 min	*DdeI*	850	600 and 250; 600, 140 and 110	[4]

References

1. K. A. Reis, B. Onal, S. Gonen, et al., “Angiotensinogen and Plasminogen Activator Inhibitor-1 Gene Polymorphism in Relation to Renovascular Disease. *CardioVascular and Interventional Radiology*, vol. 29, pp. 59-63, 2006.
2. E Martı´nez, A Puras, J Escribano, et al., “Angiotensin-converting enzyme (ACE) gene polymorphisms, serum ACE activity and blood pressure in a Spanish-Mediterranean population,” *Journal of Human Hypertension*, vol. 14, pp. 131–135, 2000.
3. M. Saeed Mahmood, K. Saboohi, S. Osman Ali, et al., “Association of the angiotensin-converting enzyme (ACE) gene G2350A dimorphism with essential hypertension,” *Journal of Human Hypertension*, vol. 17, no. 10, pp. 719-723, 2003.
4. A. V. Lapierre, M. E. Arce, J. R. Lopez, et al., “Angiotensin II type 1 receptor A1166C gene polymorphism and essential hypertension in San Luis,” *Biocell*, vol. 30, no. 3, pp. 447-455, 2006.
